# Medication utilization in traumatic brain injury patients—insights from a population-based matched cohort study

**DOI:** 10.3389/fneur.2024.1339290

**Published:** 2024-02-07

**Authors:** Yasmina Molero, David J. Sharp, Brian M. D’Onofrio, Paul Lichtenstein, Henrik Larsson, Seena Fazel, Elham Rostami

**Affiliations:** ^1^Department of Clinical Neuroscience, Centre for Psychiatry Research, Karolinska Institutet, Stockholm, Sweden; ^2^Stockholm Health Care Services, Stockholm County Council, Stockholm, Sweden; ^3^Department of Medical Epidemiology and Biostatistics, Karolinska Institutet, Stockholm, Sweden; ^4^Department of Brain Sciences, Imperial College London, London, United Kingdom; ^5^Department of Psychological and Brain Sciences, Indiana University, Bloomington, IN, United States; ^6^School of Medical Sciences, Örebro University, Örebro, Sweden; ^7^Department of Psychiatry, University of Oxford, Oxford, United Kingdom; ^8^Department of Neuroscience, Karolinska Institutet, Stockholm, Sweden; ^9^Department of Medical Sciences, Uppsala University, Uppsala, Sweden

**Keywords:** traumatic brain injury, medications, pharmacoepidemiology, sex differences, matched cohort study, population-based study

## Abstract

**Introduction:**

Traumatic brain injury (TBI) is associated with health problems across multiple domains and TBI patients are reported to have high rates of medication use. However, prior evidence is thin due to methodological limitations. Our aim was thus to examine the use of a wide spectrum of medications prescribed to address pain and somatic conditions in a population-based cohort of TBI patients, and to compare this to a sex- and age-matched cohort. We also examined how patient factors such as sex, age, and TBI severity were associated with medication use.

**Methods:**

We assessed Swedish nationwide registers to include all individuals treated for TBI in hospitals or specialist outpatient care between 2006 and 2012. We examined dispensed prescriptions for eight different non-psychotropic medication classes for the 12 months before, and 12 months after, the TBI. We applied a fixed-effects model to compare TBI patients with the matched population cohort. We also stratified TBI patients by sex, age, TBI severity and carried out comparisons using a generalized linear model.

**Results:**

We identified 239,425 individuals with an incident TBI and 239,425 matched individuals. TBI patients were more likely to use any medication [Odds ratio (OR) = 2.03, 95% Confidence Interval (CI) = 2.00–2.05], to present with polypharmacy (OR = 1.96, 95% CI = 1.90–2.02), and to use each of the eight medication classes before their TBI, as compared to the matched population cohort. Following the TBI, TBI patients were more likely to use any medication (OR = 1.83, 95% CI = 1.80–1.86), to present with polypharmacy (OR = 1.74, 95% CI = 1.67–1.80), and to use all medication classes, although differences were attenuated. However, differences increased for antibiotics/antivirals (OR = 2.02, 95% CI = 1.99–2.05) and NSAIDs/antirheumatics (OR = 1.62, 95% CI = 1.59–1.65) post-TBI. We also found that females and older patients were more likely to use medications after their TBI than males and younger patients, respectively. Patients with more severe TBIs demonstrated increased use of antibiotics/ antivirals and NSAIDs/antirheumatics than those with less severe TBIs.

**Discussion:**

Taken together, our results point to poor overall health in TBI patients, suggesting that medical follow-up should be routine, particularly in females with TBI, and include a review of medication use to address potential polypharmacy.

## 1 Introduction

Traumatic brain injury (TBI) is a leading cause of morbidity and disability across multiple domains (1), including psychiatric health, pain, cognition, and somatic complications involving cardiovascular, respiratory, endocrine, urinary, visual, and gastrointestinal systems (1–8). Consequently, individuals who sustained a TBI are reported to have high rates of medication use, and studies show that 45–85% of TBI patients are prescribed psychotropic and pain medications (9–17). Less is known, however, about the use of non-psychotropic medications in TBI patients since only a small number of studies have examined this. These studies show that diuretics and medications for gastrointestinal and cardiovascular problems are the most commonly prescribed medication classes (9, 12, 14, 17), but prevalence estimates vary widely between studies; e.g., between 5 and 86% for gastrointestinal medications, and 23–40% for cardiovascular medications (9, 12, 14).

TBI patients are also reported to have increased rates of polypharmacy, i.e., the simultaneous use of a large number of medications (9). This can be problematic, as it increases the risk for drug–drug and drug-disease interactions, and could lead to adverse events and worse recovery (9). Still, there is limited knowledge on the extent of polypharmacy in TBI patients due to the inclusion of small and selected clinical samples. There is also limited knowledge on how patient factors such as sex, age, or injury severity are associated with medication use after a TBI. Two studies have pointed to differences in medication use by age and sex (9, 17). However, studies were small which could result in greater differences in comparisons, potentially due to a more biased representation of the population at large (18).

Moreover, studies have not assessed pre-injury medication use, which is a major confound as health problems and healthcare utilization before the TBI are common (19, 20). TBI patients are also a heterogenous group that receive treatment in a variety of settings depending on the nature of their TBI sequelae, but previous studies have mainly included patients with more severe TBIs from specialized settings (e.g., rehabilitation centers) (9, 12, 14, 17). This could result in selection bias and limit the generalizability of findings, since the vast majority (70–90%) of TBIs are mild (1). Furthermore, most studies have lacked a control group of individuals without TBI, limiting the understanding of how medication use patterns may differ from the general population. Another limitation in previous research is the use of retrospective self-reports for assessing medication use, which may be subject to recall bias.

Further research using large representative samples is therefore needed. A thorough examination of medication use in TBI patients, both before and after their injury, is crucial for understanding treatment patterns and potential health implications for patient outcomes. Dispensed medications (i.e., medications collected by the patient at the pharmacy) can be used as a proxy for health conditions, and unlike patient register data in Sweden, which only includes hospitalizations and visits to open specialized care, dispensed medications capture prescriptions initiated within a variety of settings (including private and primary care). Knowledge on pre-and post-TBI medication use can thus inform healthcare providers about broader health concerns in TBI patients. This information is valuable for optimizing medication management strategies, improving long-term care planning, and tailoring interventions to address non-neurological health problems in TBI patients. Furthermore, an assessment of medication use in TBI patients could improve the knowledge-base by identifying frequently prescribed medications that would benefit from TBI patient-specific effectiveness and safety analyses, identify potential polypharmacy, and inform research design by providing knowledge on how important patient factors (such as sex, age, or injury severity) are associated with post-TBI medication use (10). To our knowledge, no study has examined non-psychotropic medications in a nationwide cohort of TBI patients, assessed pre-injury medication use, or included a matched population cohort as a comparison group.

The aim of the current study was to examine the use of a wide spectrum of non-psychotropic medications prescribed to address pain and somatic complications in a population-based cohort of TBI patients during the 12 months before, and 12 months after their TBI, and compare this to a matched population cohort. We also examined how patient factors such as sex, age, and TBI severity were associated with post-TBI medication use in TBI patients.

## 2 Materials and methods

### 2.1 Ethics

The project follows the Declaration of Helsinki and was approved by the Swedish Ethical Review Authority (2013/862–31/5), which waived the need for informed consent due to the register-based design.

### 2.2 Setting and study period

We used Swedish registers with nationwide coverage that were linked through each individual’s identification number (21). All data were pseudonymized. The start of study period was July 1, 2005, and the end of the study period was December 31, 2013. The study period was defined according to the data available on medications; we examined medication use 12 months prior to the TBI, and the Swedish Prescribed Drug Register started in July 2005. The data linkage included data until December 2013.

### 2.3 Study design and participants

This is a matched population cohort study. TBI patients included all individuals aged 18 and over who were treated for TBI in a hospital or specialized open care between July 1, 2006, and December 31, 2012, to allow for examination of medication use during the 12 months before, and 12 months after, the TBI date. We also included a general population cohort that was matched to each TBI patient on sex and birthyear (1:1 match). Individuals in the matched population cohort were alive and living in Sweden at the date of their matched TBI patient’s TBI date and had not been diagnosed with TBI before December 31, 2013.

### 2.4 Measures

#### 2.4.1 TBI

We used the Centers for Disease Control and Prevention definition of TBI (22) (International Classification of Diseases, 10th revision [ICD-10]: S01.0–S01.9, S02.0, S02.1, S02.3, S02.7–S02.9, S04.0, S06.0–S06.9, S07.0, S07.1, S07.8. S07.9, S09.7–S09.9, T01.0, T02.0, T04.0, T06.0, T90.1, T90.2, T90.4, T90.5, T90.8, T90.9). We included only the incident (i.e., first) TBI diagnosis, thus excluding all individuals who had been diagnosed with TBI before the start of the study period (ICD-9: 800–804, 851–854; ICD-10: as above). Information on ICD-9/10 TBI diagnoses was collected from the Swedish Patient Register (23), which includes all admissions to hospitals and outpatient contacts with specialized open care (including visits to the emergency department). This register has excellent validity on inpatient treatment for ICD-10 TBIs (sensitivity = 95–97%; specificity = 96–98%) (24). However, TBI diagnoses made in specialized outpatient care have not been validated. Missing data in The Swedish Patient Register is around 1% for inpatient treatment, and around 3% for outpatient treatment (23).

#### 2.4.2 Medications

Information was extracted from the Swedish Prescribed Drug Register, which includes information on all prescriptions that have been collected at all pharmacies in Sweden (with less than 0.3% missing information) (25). We classified medications by the Anatomical Therapeutic Chemical (ATC) code that is a globally recognized system for classifying and categorizing pharmaceutical substances based on their therapeutic and chemical properties. We examined a wide spectrum of non-psychotropic medications prescribed to address pain and somatic complications involving cardiovascular, respiratory, endocrine, urinary, visual, and gastrointestinal systems (1–8). Medications included gastrointestinal and diabetes medications (ATC: A01, A02, A07, A10), cardiovascular medications (ATC: C01–C03, C05, C07–C10), genito-urinary medications and sex hormones (ATC: G02–G04), systemic hormonal preparations (ATC: H01–H05), antibiotics and antivirals (ATC: J01, J02, J04, J05), Non-steroidal anti-inflammatory drugs (NSAIDs) and antirheumatics (ATC: M01), respiratory system agents (ATC: R01, R03, R05, R06), and eye medications (ATC: S01). We defined polypharmacy as the presence of five or more different medication classes during 1 year (26).

#### 2.4.3 Demographic measures

Information on sex and age was collected from the Total Population Register (21).

#### 2.4.4 TBI severity

TBI severity was measured in two ways: (1) Receiving inpatient treatment (i.e., being hospitalized for the TBI) vs. receiving outpatient treatment (i.e., treated only in specialized open care) and; (2) Presenting with polytrauma (i.e., having a co-occurring injury to another body part or system on the same day as the TBI; ICD-10: S00-S99, T00-T19, T90-T98, excluding TBI diagnoses) vs. presenting with TBI only.

#### 2.4.5 Diagnosed disorders

Information on diagnosed disorders for the 12 months before (up until the day before the TBI) and the 12 months after the TBI date (starting on the day of the TBI), was collected from the Swedish Patient Register. This included ICD-10 diagnoses recorded during admissions to hospitals and outpatient contacts with specialized open care; psychiatric disorders (F20–F99), substance use disorders (F10–F16, F18–F19), dementia (F00–F03), stroke (I60–I64), epilepsy (G40–G41), sleep disorders (G47), other neurological conditions (A80–A89, G00–G26, G35–G37, G46, G91, I65–I69), cardiovascular disorders (I05–I15, I20–I28, I30–I52, I70–I79), endocrine and metabolic disorders (E00–E07, E10–E16, E20–E35, O24), and gastrointestinal disorders (K25–K31, K50–K51, K70–K77, K80–K85, K90).

### 2.5 Statistical analyses

We measured the use of any medication (i.e., having collected at least one medication), polypharmacy (i.e., five or more different medication classes), and each of the eight medication classes in TBI patients and the matched population cohort. We divided medication periods into the 12 months before (up until the day before the TBI) and the 12 months after the TBI date (starting on the day of the TBI). When examining prevalence rates, we stratified medication use in the 12 months after the TBI date into two categories: (a) new use (i.e., the first collected prescription during the 24-month study period, was after the TBI date), and (b) prevalent use (i.e., a prescription had also been collected in the 12 months prior to the TBI date). For the matched population cohort, we measured the same time-period as their matched TBI patient.

We estimated the odds ratio (OR) of medication use in TBI patients as compared to the matched population cohort by applying a fixed-effects model using conditional logistic regression (27), where each matched pair was considered a stratum (more details in Supplementary material). This approach allowed us to estimate the OR for medication use in TBI patients relative to the matched population cohort, while controlling for the matched structure (i.e., within-pair variability) of the data. For this analysis, we ran the PROC LOGISTIC procedure in SAS 9.4, using the STRATA statement. In the analyses of post-TBI medication use, the model was adjusted for pre-TBI medication use within the same category (e.g., in the analyses of post-TBI cardiovascular medication use, the model was adjusted for pre-TBI cardiovascular medication use) to account for use that was initiated before the TBI.

To examine how patient- and injury-specific factors were associated with post-TBI medication use, we examined TBI patients only. We performed a generalized linear model (GLM) analysis where the predictor variable was patient sex (female patient vs. male patient) or TBI severity (inpatient vs. outpatient, and polytrauma vs. TBI only), respectively. The response variable was the binary outcome of post-TBI medication use. We ran the PROC GENMOD procedure in SAS 9.4 using a Poisson distribution with a robust variance estimator and log link function. The GLM provides estimates of the risk ratio (RR) on the association between each predictor variable (e.g., sex) and post-TBI medication use (more details in Supplementary material). First, we compared post-TBI medication use (i.e., during the 12 months following the TBI) in female TBI patients as compared to male TBI patients. This model was adjusted for age (as a continuous covariate) and pre-TBI medication use (i.e., use of the medication during the 12 months leading up to the TBI). Second, we investigated if post-TBI medication use varied by TBI severity. We performed two separate GLM analyses for this: (1) We estimated the RR of post-TBI medication use in TBI patients who received inpatient treatment (i.e., were hospitalized) as compared to TBI patients who received outpatient treatment (i.e., specialized open care) and (2) We estimated the RR of post-TBI medication use in TBI patients with polytrauma (i.e., TBI and at least one co-occurring physical injury) as compared to TBI patients without co-occurring physical injuries. Both models were adjusted for sex, age (as a continuous covariate), and pre-TBI medication use.

### 2.6 Sensitivity analyses

Because age is a strong prognostic factor for negative outcomes after a TBI (28), and older age has been associated with higher medication use after a TBI (9), we carried out sensitivity analyses where we stratified TBI patients by age at injury. We stratified them into four pre-specified age categories; ages 18–30, 31–50, 51–70, and 71 and older, at the time of their TBI. We then examined if post-TBI medication use varied by age by performing a GLM analysis to estimate the RR for medication use in each of the older age categories as compared to TBI patients aged 18–30. The model was adjusted for sex and pre-TBI medication use.

All results are presented with 95% Confidence Intervals (CIs). We followed the Strengthening the Reporting of Observational studies in Epidemiology (STROBE) reporting guidelines for cohort studies.

## 3 Results

### 3.1 Characteristics of TBI patients and matched population cohort

We identified 239,425 individuals aged 18 and over who had been treated for an incident TBI in a hospital or specialist outpatient care between July 1, 2006, and December 31, 2012 (Table 1). TBI patients included 41.1% females and 58.8% males, and males were on average younger than females at the date of the TBI (median age for males = 45 years and females =59 years; Supplementary Table S1). Furthermore, 27.0% received inpatient treatment for their TBI and 18.6% presented with polytrauma, i.e., had a co-occurring body injury in addition to the TBI. We matched each TBI patient to an individual in the general population on age and sex (*n* = 239,425).

**Table 1:** Demographic and health characteristics of individuals with TBI and matched population cohort.

	TBI patients (*n* = 239,425)	Matched population cohort (*n* = 239,425)
Age at incident TBI		
18–30	26.8% (64,115)	26.8% (64,115)
31–50	23.2% (55,624)	23.2% (55,624)
51–70	23.2% (55,474)	23.2% (55,474)
71 and older	26.8% (64,212)	26.8% (64,212)
Median age (IQR)	51 (30, 73)	51 (30, 73)
Sex		
Women	41.2% (98,532)	41.2% (98,532)
Men	58.8% (140,986)	58.8% (140,986)
Incident TBI characteristics		
Inpatient treatment	27.0% (64,777)	–
Polytrauma	18.6% (44,509)	–
Diagnoses 12 months before incident TBI		
Psychiatric disorders	5.7% (13,629)	2.0% (4,820)
Substance use disorders	3.3% (7,892)	0.4% (946)
Dementia	1.6% (3,902)	0.3% (723)
Stroke	2.1% (5,076)	0.4% (950)
Epilepsy	1.2% (2,742)	0.2% (562)
Sleep disorders	0.5% (1,084)	0.3% (650)
Other neurological conditions	2.7% (6,480)	0.8% (1,870)
Cardiovascular diseases	11.0% (26,420)	5.5% (13,082)
Endocrine and metabolic disorders	4.7% (11,274)	2.5% (5,971)
Gastrointestinal disorders	2.3% (5,556)	1.2% (2,905)
Diagnoses 12 months after incident TBI		
Psychiatric disorders	6.9% (16,411)	2.1% (4,991)
Substance use disorders	6.2% (14,900)	0.4% (979)
Dementia	3.3% (7,834)	0.4% (898)
Stroke	3.6% (8,589)	0.4% (1,015)
Epilepsy	2.0% (4,688)	0.3% (602)
Sleep disorders	0.6% (1,351)	0.3% (661)
Other neurological conditions	4.7% (11,187)	0.9% (2,172)
Cardiovascular diseases	17.7% (42,272)	6.1% (14,545)
Endocrine and metabolic disorders	6.8% (16,311)	2.7% (6,389)
Gastrointestinal disorders	2.6% (6,272)	1.3% (3,051)

For matched population cohort, 12-month prevalence of diagnoses was calculated from the same TBI date as their corresponding TBI match; Cardiovascular diseases excludes cerebrovascular diseases.

The most common pre-TBI diagnosed disorders were cardiovascular diseases, endocrine and metabolic disorders, and psychiatric disorders (Table 1). During the year after the TBI, rates of all investigated disorders increased in TBI patients. Individuals in the matched population cohort demonstrated lower prevalence rates of all disorders in the 12 months leading up to the TBI date (i.e., the date of their matched TBI patient). In the 12 months after the TBI date, prevalence rates in the matched cohort remained similar for most disorders with the exception of cardiovascular diseases, which increased from 5.5 to 6.1%.

### 3.2 Medication use during the 12 months before and 12 months after the TBI

**Figure 1:**
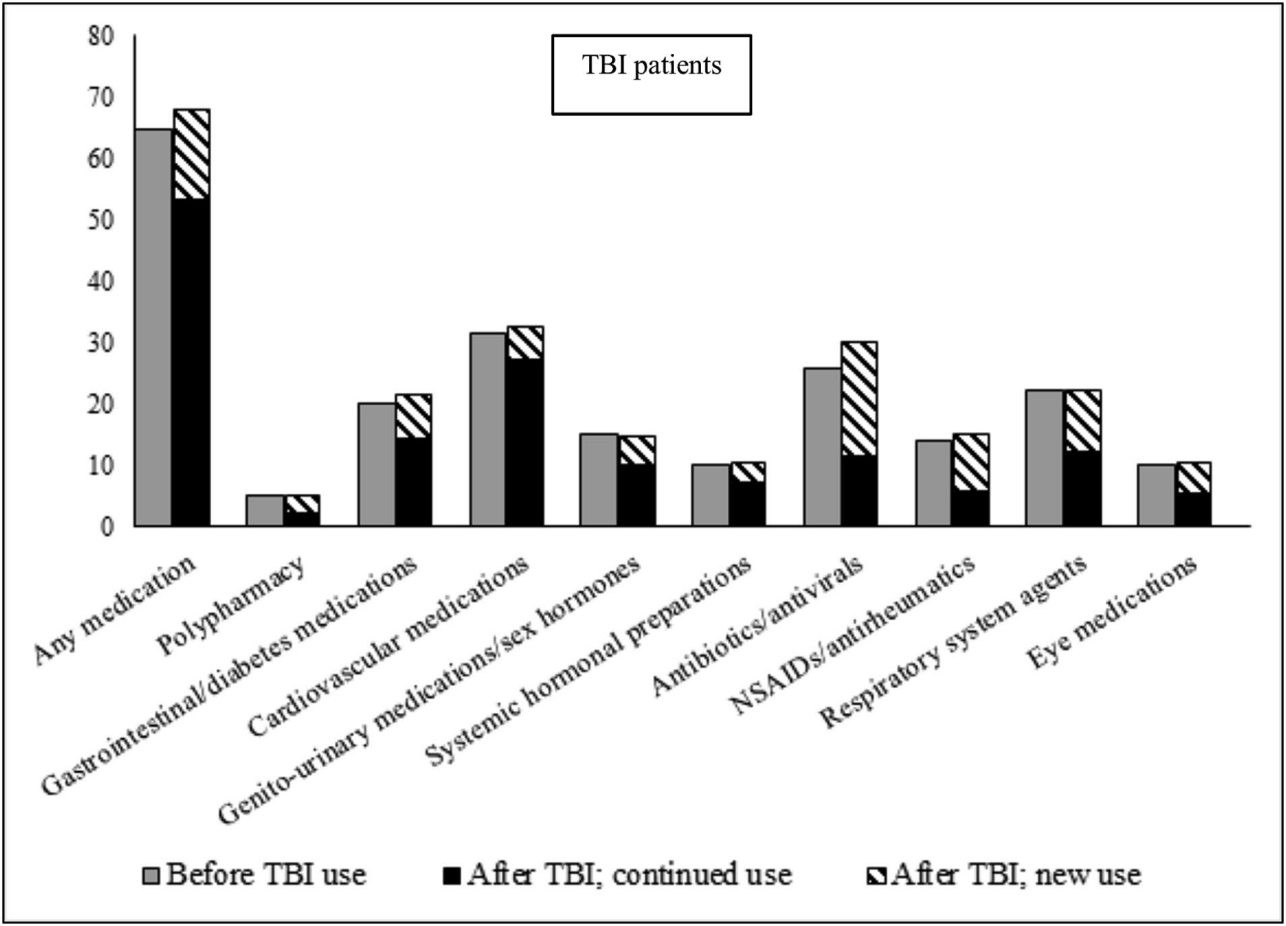
Prevalence of medication use during 12 months before, and 12 months after (stratified by continued use and new use), TBI date in TBI patients. Continued use, Used the medication during the 12 months prior to the TBI; New use, Did not use the medication during the 12 months prior to the TBI; NSAIDs, Non-steroidal anti-inflammatory drugs.

**Figure 2:**
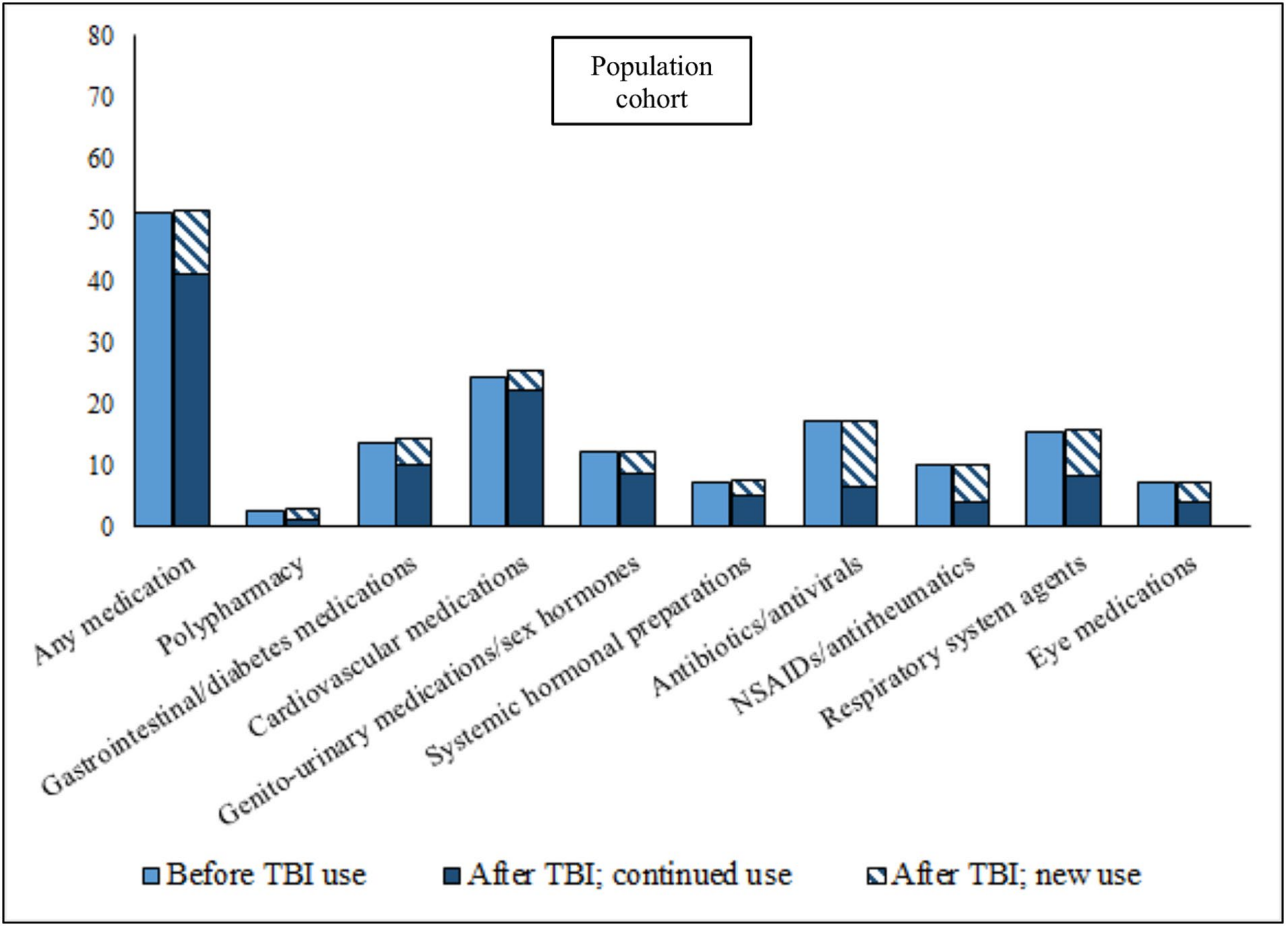
Prevalence of medication use during 12 months before, and 12 months after (stratified by continued use and new use), TBI date in matched population cohort. Continued use, Used the medication during the 12 months prior to the TBI; New use, Did not use the medication during the 12 months prior to the TBI; NSAIDs, Non-steroidal anti-inflammatory drugs.

In the 12 months leading up to the TBI date, 64.6% of TBI patients had collected a prescription for at least one of the eight medication classes studied, as compared to 51.0% of the population cohort (Figures 1, 2; prevalence rates and details of specific medications within each class in Supplementary Tables S2, S3. Prevalence rates stratified by sex and age categories provided in Supplementary Tables S4, S6, respectively). TBI patients also displayed higher prevalence rates of polypharmacy (i.e., they had collected prescriptions for five or more different medication classes during the 12-month time-period), and of each of the eight medication classes. In the 12 months after the TBI date, prevalence rates of all medications increased slightly in TBI patients, particularly for antibiotics/antivirals (an increase from 25.9% pre-TBI to 30.0% post-TBI). Prevalence rates remained similar in the population cohort in the 12 months after. We also divided post-TBI medication use into two categories; new use (i.e., where the first collected prescription during the 24-month study period was after the TBI date), and prevalent use (i.e., a prescription had also been collected in the 12 months prior to the TBI date). For TBI patients, the largest increases in new use were seen for antibiotics/antivirals, and NSAIDs/antirheumatics, where two-thirds of post-TBI prescriptions were new. The matched cohort presented similar rates in new use.

### 3.3 Medication use in TBI patients as compared to matched population cohort

**Figure 3:**
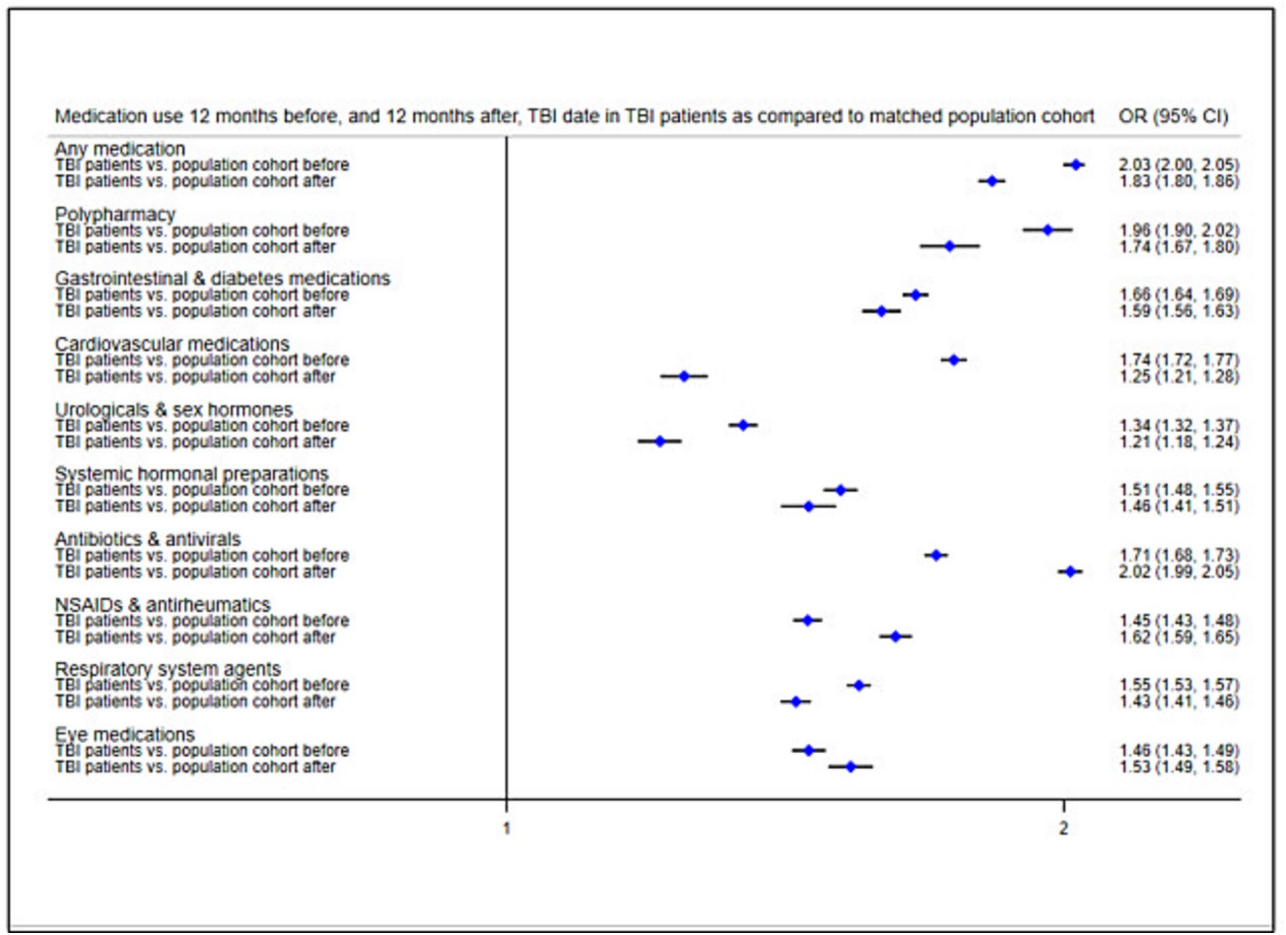
Odds ratios of medication use 12 months before, and 21 months after, TBI date in TBI patients as compared to the matched population cohort. Reference group, Matched population cohort; OR, Odds ratio; NSAIDs, Non-steroidal anti-inflammatory drugs.

We applied a fixed-effects model using conditional logistic regression to compare medication use in TBI patients with that in the matched population cohort (Figure 3; prevalence rates in Supplementary Table S3). In the 12 months before the TBI date, TBI patients were around twice as likely to collect any medication (OR = 2.03, 95% CI = 2.00–2.05) and to present with polypharmacy (OR = 1.96, 95% CI = 1.90–2.02) than the matched population cohort. TBI patients demonstrated increased ORs for each of the eight medication classes before the TBI date (ranging between 1.34 for genito-urinary medications/sex hormones and 1.74 for cardiovascular medications). We repeated the same analyses to compare medication use in TBI patients to the matched population cohort in the 12 months after the TBI date, while also adjusting for previous medication use (i.e., in the 12 months leading up to the TBI date). Results showed attenuated ORs for any medication (OR = 1.83, 95% CI = 1.80–1.86), polypharmacy (OR = 1.74, 95% CI = 1.57–1.80), and each of the medication classes (ranging between 1.21 for genito-urinary medications/sex hormones and 1.59 for gastrointestinal/diabetes medications). However, ORs increased after the TBI for antibiotics/antivirals (OR = 2.02, 95% CI = 1.99–2.05), NSAIDs/antirheumatics (OR = 1.62, 95% CI = 1.59–1.65), and eye medications (OR = 1.53, 95% CI = 1.49–1.58).

### 3.4 Post-TBI medication use in TBI patients by sex

**Figure 4:**
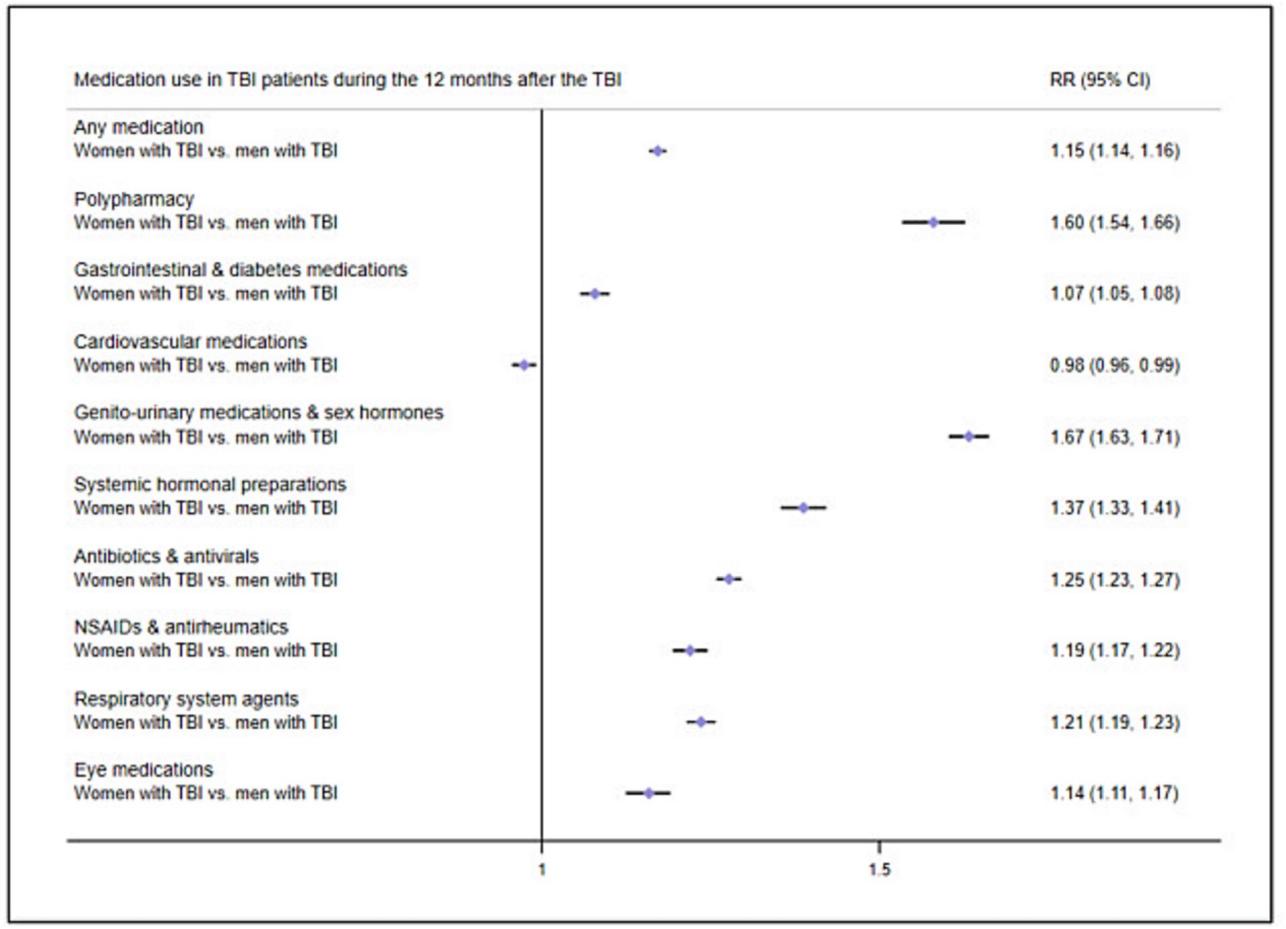
Risk ratios of medication use in TBI patients 12 months after the TBI by sex. Reference group, Men with TBI; RR, Risk ratio; NSAIDs, Non-steroidal anti-inflammatory drugs.

We then examined how patient factors were associated with post-TBI medication use by stratifying TBI patients by sex. We carried out GLM analyses comparing post-TBI medication use in female TBI patients to that male TBI patients, while adjusting for age and previous medication use. During the 12 months after the TBI, female TBI patients presented higher RRs of collecting any medication (RR = 1.15, 95% CI = 1.14–1.16) and of polypharmacy (RR = 1.60, 95% CI = 1.54–1.66) as compared to male TBI patients (Figure 4; prevalence rates in Supplementary Table 4). Female TBI patients also presented increased RRs for all medication classes (ranging between 1.07 and 1.67) except for cardiovascular medications.

### 3.5 Post-TBI medication use in TBI patients by TBI severity

**Figure 5:**
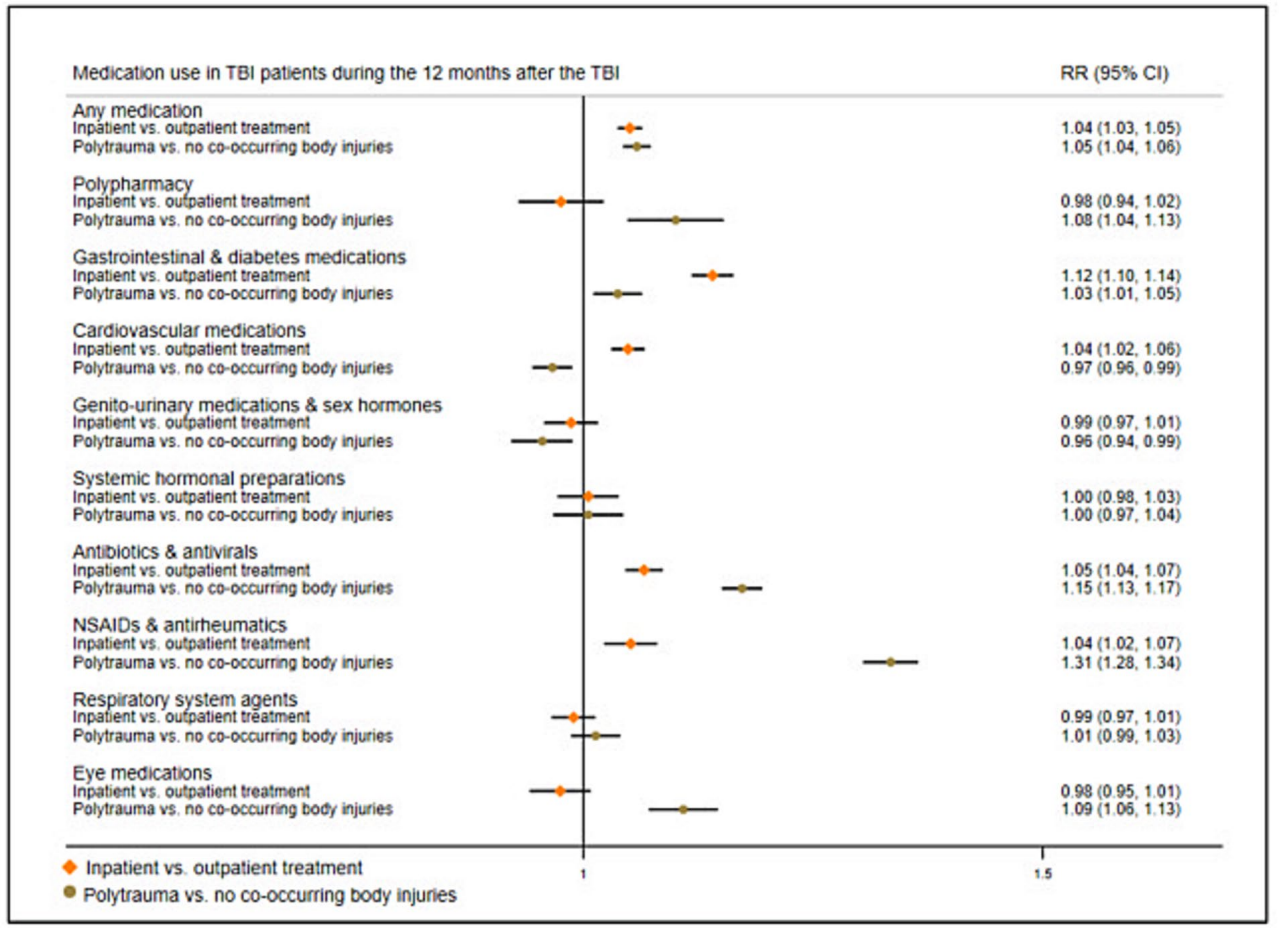
Risk ratios of medication use in TBI patients 12 months after the TBI by injury severity. Reference group, TBI patients who received outpatient treatment, and TBI patients with no co-occurring body injuries, respectively; RR, Risk ratio; NSAIDs, Non-steroidal anti-inflammatory drugs.

We also examined how patient factors were associated with post-TBI medication use by stratifying TBI patients by injury severity. We carried out GLM analyses comparing post-TBI medication use in those with more severe injuries to those with less severe injuries (Figure 5; prevalence rates in Supplementary Table S5). First, we compared individuals who had been hospitalized for their TBI to those who received outpatient treatment only. Second, we compared individuals with polytrauma (i.e., TBI plus body injury) to those with only TBI. Models were adjusted for sex, age, and previous medication use. Results from both models showed some similarities; during the 12 months after the TBI, individuals with more severe injuries (i.e., hospitalized individuals and individuals with polytrauma) demonstrated increased RRs of collecting gastrointestinal/diabetes medications, antibiotics/antivirals, and NSAIDs/antirheumatics.

### 3.6 Sensitivity analyses—post-TBI medication use in TBI patients by age category

In sensitivity analyses, we examined how age was associated with post-TBI medication use. We carried out a GLM comparing medication use in TBI patients in different age categories; we compared TBI patients aged 31–50, 51–70, and 71 and older, respectively, to those aged 18–30 (Supplementary Figure S1; prevalence rates in Supplementary Table S6). Analyses were adjusted for sex and previous medication use. Results showed that during the 12 months after the TBI, individuals in the older age categories presented increased RRs of medication use (ranging between 1.14 and 8.36), and the general pattern was that RRs increased with each increasing age category.

## 4 Discussion

In a nationwide Swedish study, we identified a cohort of 239,425 individuals treated for an incident (i.e., first) TBI and matched them to 239,425 individuals in the general population who had not been treated for TBI. We examined the use of eight different non-psychotropic medication classes for treating pain and somatic complications. We found that TBI patients were twice as likely to use any medication in the 12 months leading up to the TBI date, as compared to the matched population cohort. They were also more likely to use each of the eight medication classes and to present with polypharmacy before their TBI. In the 12 months following the TBI, we found the largest increases in new use (i.e., medication use initiated after the TBI) for antibiotics/antivirals and NSAIDs/antirheumatics, where around two-thirds of prescriptions were new. We also found that TBI patients continued to be more likely to use all medications and to present with polypharmacy as compared to the matched population cohort post-TBI, although differences were attenuated. However, for antibiotics/antivirals, NSAIDs/antirheumatics, and eye medications, differences between TBI patients and the matched cohort increased after the TBI. We also examined how patient factors such as sex, age, and TBI severity were associated with post-TBI medication use in TBI patients. We found that female patients were more likely than male patients to use medications after their TBI, and that older patients were more likely than younger patients after their TBI. Our results also showed that patients with more severe TBIs demonstrated increased use of certain medications (i.e., gastrointestinal/diabetes medications, antibiotics/antivirals, and NSAIDs/antirheumatics) than those with less severe TBIs.

Our results on pre- and post-TBI medication use are in line with our previous study of the same cohort, where we showed that TBI patients had increased rates of psychotropic and pain medication use, both in the 12 months before and in the 12 months after their TBI (29). In fact, we found only a slight increase in new use (i.e., use initiated after the TBI) for most non-psychotropic medications. The highest rates in new use were shown for antibiotics/antivirals and NSAIDs/antirheumatics, which could be due to penetrating wounds, injury complications, and/or increased pain after the TBI (30–32). These findings add to the growing body of evidence suggesting that health events precede the TBI (19, 20), and could have implications for TBI prevention as well as clinical management after the TBI (33). Prevalence rates in our study were on the lower end of previously reported ranges; for example, we found that 32.5% were prescribed a cardiovascular medication (reported range: 5–86%) and 21.5% were prescribed a gastrointestinal/diabetes medication (reported range: 23–40%) (9, 12, 14). While previous studies mainly included patients in rehabilitation centers with severe injuries, we included a population-based sample where the majority (73%) were not hospitalized for their TBI, which could explain the lower prevalence rates in our study. Differences between studies could also be due to differing sources of prescription information, varying lengths of follow-up, and/or to our study period (2005–2013). Our data cutoff in December 31, 2013 could affect the generalizability of our results to current patient cohorts if prescription practices for the studied medications changed since the study period. However, no fundamental reorganization or new policy has been adapted in Sweden regarding post-TBI care since this period, and no groundbreaking medications have been introduced for TBI patients. Although there have been changes in general prescription patterns for certain drugs used in other medical conditions, such as antibiotic regimes, diabetes, and cardiovascular conditions, our analysis is based on the group and subgroup of drugs classified according to the Anatomical Therapeutic Chemical (ATC) code. Consequently, changes in prescription practices from one medication to another within the same class/group do not affect our estimates. Therefore, the results from our study should remain relevant and generalizable to more recent TBI patients. Furthermore, the most commonly dispensed medication class in our study was cardiovascular medications, in line with previous research (12, 14, 17), and suggesting consistency with newer samples.

We also found higher rates of polypharmacy in TBI patients as compared to the matched population cohort, both before and after the TBI. Although differences were reduced after the TBI (from OR = 1.96, 95% CI = 1.90–2.02 to OR = 1.74, 95% CI = 1.67–1.80), TBI patients still presented an increased risk of polypharmacy after adjustments for age, sex, and pre-TBI medication use. TBI patients may experience a variety of physical health problems after their injury, including motor impairment, chronic pain, hormonal imbalance, cardiovascular conditions, digestive issues, and sleep disturbances (1–8), that may lead to an overall decline in function. Management of these problems could lead to the unintended use of multiple medications due to separate treatment settings and guidelines (34). However, polypharmacy is of concern as it may affect medication effectiveness and safety, raise the risk of drug–drug interactions, and increase mortality after the TBI (35–37). This suggests that strategies to minimize polypharmacy could be implemented in TBI patients, including central coordination, open communication and collaboration between healthcare professionals, close monitoring of potential adverse effects, and frequent reviews of medication regimens (3).

Female TBI patients in our study were, on average, older than male patients (median age 59 and 45 years, respectively), which may have affected the increased rates of pre-TBI medication use in females. However, female TBI patients also presented increased post-TBI medication use in analyses that were adjusted for age and pre-TBI medication use, which could suggest more morbidity in females after the TBI. Previous studies on sex differences in TBI outcomes have been inconclusive; some studies suggest that females have worse outcomes in a wide range of areas (18), which has been attributed to differences in hormonal and chromosomal factors (38). It has also been suggested that females may report more symptoms after their TBI as it is more socially acceptable for them to admit health problems (38). Our results may thus reflect sex differences in acknowledging health problems and seeking treatment. Nevertheless, female patients are underrepresented in TBI research (38), pointing to a need for more research to examine potential sex differences in TBI outcomes.

TBI patients in our study showed higher rates of diagnosed substance use disorders than the matched population, and rates were almost doubled in the year following the TBI; from 3.3 to 6.2%. Previous studies have reported a high prevalence of pre-injury substance misuse in TBI patients, that in many cases contributed to the TBI (39). It has also been suggested that TBI increases the risk of developing subsequent misuse problems due to neurobiological damage, TBI sequelae (e.g., poor emotional regulation), or maladaptive ways to handle stress or pain stemming from the TBI (40–42). The rise in diagnosed substance use disorders after the TBI in our study may also be influenced by detection bias. This could occur if pre-injury disorders were identified post-injury due to intoxication at the time of the TBI, or increased healthcare contacts post-TBI that affected the likelihood of detection and diagnosis. Nonetheless, substance use disorders are associated with negative health effects, e.g., cardiovascular or liver diseases (43), which could affect medication rates in the TBI cohort.

## 5 Strengths and limitations

This study has several strengths; we included all individuals treated for TBI in Sweden during the study period and linked several nationwide registers. Our information on medications was based on individuals collecting their medication from pharmacies, an advance from prescription-only data and self-reports, and data was nearly complete (less than 0.3% missing information) (25). We included medications prescribed in all healthcare settings, i.e., hospitals, rehabilitation centers, open specialized care, and primary care and compared medication use to a population cohort matched on sex and age. Several limitations should be considered; we only examined medications collected at pharmacies, as medications dispensed in hospitals are not available in the Swedish Prescribed Drug Register. For individuals with extended hospital stays, this could lead to an underestimation of medication use. However, the majority of TBI patients (73%) were not admitted overnight, and 99.8% (*n* = 238,715) were discharged within 30 days. Furthermore, we examined collected medications, and had no information on medication adherence. However, collected medications reflect the health problems they were prescribed to address. We could not include patients from the last 10 years due to data availability, which could affect the generalizability of results. Nonetheless, post-TBI treatment strategies in Sweden have remained largely unchanged since the end of our study period in 2013. We also lacked information on anticoagulants, which have been linked to poorer outcomes in TBI patients (44). TBI diagnoses were collected from the Swedish Patient Register, which includes all disorders diagnosed in hospitals and specialized outpatient care, and TBI diagnoses made solely in primary care were not captured. This likely underestimated rates of mild TBIs in the population. We lacked detailed clinical data for the classification of TBI severity (e.g., the Glasgow Coma Scale), but we used other proxies for measuring injury severity, such as hospitalization and polytrauma. Another limitation included the lack of information on the clinical severity of somatic illness, both before and after the TBI. There is a research gap on the severity of somatic illness in TBI patients, and future research should address this gap and its implications in patients with TBI. Moreover, differences between countries in prescription practices or service provision may affect the generalizability of findings. Rates of TBI-related hospital discharges are higher in Sweden as compared to the European average (age-adjusted rate per 100,000 individuals: Sweden 445.8; Europe 287.2) (45), although this could be due to between-country differences in data collection and coding.

## 6 Conclusion

Our findings showed that individuals who sustained a TBI had a greater likelihood of being prescribed non-psychotropic medications and of polypharmacy, before and after their TBI, than a sex- and age-matched population cohort. These results are in line with previous work on psychotropic and pain medications showing higher medication use in TBI patients both before and after their TBI (29). This suggests that health problems precede the TBI, which could have implications for post-TBI clinical care. Our findings also suggested that female TBI patients were more likely to use medications than their male counterparts. Taken together, these results point to poor overall health in TBI patients, which is a barrier to social participation and negatively influences employment, quality of life, and level of independence (3). This suggests a need for addressing psychiatric and non-neurological symptoms in TBI care, particularly in females with TBI, and to review the use of multiple medications to address potential polypharmacy.

## Data availability statement

The datasets presented in this article are not readily available because data may be obtained from a third party and are not publicly available. The Public Access to Information and Secrecy Act in Sweden prohibits us from making individual level data publicly available due to ethical concerns about identification. Researchers who are interested in replicating our work can apply for individual level data from: Statistics Sweden (mikrodata@scb.se) for data from the Total Population Register; the National Board of Health and Welfare (registerservice@socialstyrelsen.se) for data from the Patient Register, the Prescribed Drug Register, and the Cause of Death Register. Requests to access the datasets should be directed to mikrodata@scb.se; registerservice@socialstyrelsen.se.

## Ethics statement

The studies involving humans were approved by the Swedish Ethical Review Authority. The studies were conducted in accordance with the local legislation and institutional requirements. The ethics committee/institutional review board waived the requirement of written informed consent for participation from the participants or the participants’ legal guardians/next of kin because the need for informed consent was waived due to the register-based design.

## Author contributions

YM: Conceptualization, Data curation, Formal analysis, Funding acquisition, Investigation, Methodology, Project administration, Validation, Visualization, Writing – original draft, Writing – review & editing. DS: Conceptualization, Writing – review & editing. BD’O: Conceptualization, Funding acquisition, Resources, Writing – review & editing. PL: Conceptualization, Funding acquisition, Resources, Writing – review & editing. HL: Conceptualization, Funding acquisition, Resources, Writing – review & editing. SF: Conceptualization, Writing – review & editing. ER: Conceptualization, Methodology, Supervision, Writing – original draft, Writing – review & editing.

## References

[1] Maas AIR Menon DK Adelson PD Andelic N Bell MJ Belli A. Traumatic brain injury: integrated approaches to improve prevention, clinical care, and research. Lancet Neurol. (2017) 16:987–1048. doi: 10.1016/s1474-4422(17)30371-x, PMID: 29122524

[2] Bhatnagar S Iaccarino MA Zafonte R. Pharmacotherapy in rehabilitation of post-acute traumatic brain injury. Brain Res. (2016) 1640:164–79. doi: 10.1016/j.brainres.2016.01.021, PMID: 26801831

[3] Murphy MP Carmine H. Long-term health implications of individuals with TBI: a rehabilitation perspective. Neurorehabilitation. (2012) 31:85–94. doi: 10.3233/nre-2012-0777, PMID: 22523016

[4] Colantonio A. Sex, gender, and traumatic brain injury: a commentary. Arch Phys Med Rehabil. (2016) 97:S1–4. doi: 10.1016/j.apmr.2015.12.002, PMID: 26804988

[5] Katzenberger RJ Ganetzky B Wassarman DA. The gut reaction to traumatic brain injury. Fly. (2015) 9:68–74. doi: 10.1080/19336934.2015.1085623, PMID: 26291482 PMC5019014

[6] Howlett JR Nelson LD Stein MB. Mental health consequences of traumatic brain injury. Biol Psychiatry. (2021) 91:413–20. doi: 10.1016/j.biopsych.2021.09.024, PMID: 34893317 PMC8849136

[7] Mahajan C Prabhakar H Bilotta F. Endocrine dysfunction after traumatic brain injury: an ignored clinical syndrome? Neurocrit Care. (2023) 39:714–23. doi: 10.1007/s12028-022-01672-3, PMID: 36788181 PMC10689524

[8] Armstrong RA. Visual problems associated with traumatic brain injury. Clin Exp Optom. (2018) 101:716–26. doi: 10.1111/cxo.12670 29488253

[9] Cosano G Giangreco M Ussai S Giorgini T Biasutti E Barbone F. Polypharmacy and the use of medications in inpatients with acquired brain injury during post-acute rehabilitation: a cross-sectional study. Brain Inj. (2016) 30:353–62. doi: 10.3109/02699052.2015.1118767 26890986

[10] Hammond FM Barrett RS Shea T Seel RT McAlister TW Kaelin D. Psychotropic medication use during inpatient rehabilitation for traumatic brain injury. Arch Phys Med Rehabil. (2015) 96:S256–3.e14. doi: 10.1016/j.apmr.2015.01.025, PMID: 26212402 PMC4516906

[11] Lauterbach MD Notarangelo PL Nichols SJ Lane KS Koliatsos VE. Diagnostic and treatment challenges in traumatic brain injury patients with severe neuropsychiatric symptoms: insights into psychiatric practice. Neuropsychiatr Dis Treat. (2015) 11:1601–7. doi: 10.2147/ndt.s80457, PMID: 26170672 PMC4494623

[12] Yasseen B Colantonio A Ratcliff G. Prescription medication use in persons many years following traumatic brain injury. Brain Inj. (2008) 22:752–7. doi: 10.1080/02699050802320132, PMID: 18787984

[13] Starosta AJ Adams RS Marwitz JH Kreutzer J Monden KR Dams O'Connor K. Scoping review of opioid use after traumatic brain injury. J Head Trauma Rehabil. (2021) 36:310–27. doi: 10.1097/htr.0000000000000721, PMID: 34489382 PMC8428300

[14] Han MH Craig SB Rutner D Kapoor N Ciuffreda KJ Suchoff IB. Medications prescribed to brain injury patients: a retrospective analysis. Optometry. (2008) 79:252–8. doi: 10.1016/j.optm.2008.01.001 18436165

[15] Vehviläinen J Skrifvars MB Reinikainen M Bendel S Marinkovic I Ala-Kokko T. Psychotropic medication use among patients with a traumatic brain injury treated in the intensive care unit: a multi-Centre observational study. Acta Neurochir. (2021) 163:2909–17. doi: 10.1007/s00701-021-04956-3, PMID: 34379205 PMC8437905

[16] Albrecht JS Mullins DC Smith GS Rao V. Psychotropic medication use among medicare beneficiaries following traumatic brain injury. Am J Geriatr Psychiatry. (2017) 25:415–24. doi: 10.1016/j.jagp.2016.11.018, PMID: 28111062 PMC5365362

[17] Merino R Pérez A Fierro J Terré R. Prevalence of medication and off-label medication use in acquired brain injury at a neurorehabilitation hospital. Eur J Clin Pharmacol. (2019) 75:985–94. doi: 10.1007/s00228-019-02651-y, PMID: 30834963

[18] Gupte R Brooks W Vukas R Pierce J Harris J. Sex differences in braumatic brain injury: what we know and what we should know. J Neurotrauma. (2019) 36:3063–91. doi: 10.1089/neu.2018.6171, PMID: 30794028 PMC6818488

[19] Dell KC Grossner EC Staph J Schatz P Hillary FG. A population-based study of pre-existing health conditions in traumatic brain injury. Neurotrauma Rep. (2021) 2:255–69. doi: 10.1089/neur.2020.0065, PMID: 34223556 PMC8244518

[20] Albrecht JS Wickwire EM. Healthcare utilization following traumatic brain injury in a large national sample. J Head Trauma Rehabil. (2021) 36:E147–54. doi: 10.1097/htr.0000000000000625, PMID: 33201034

[21] Ludvigsson JF Almqvist C Bonamy AK Ljung R Michaëlsson K Neovius M. Registers of the Swedish total population and their use in medical research. Eur J Epidemiol. (2016) 31:125–36. doi: 10.1007/s10654-016-0117-y 26769609

[22] Coronado VG Xu L Basavaraju SV McGuire LC Wald MM Faul MD. Surveillance for traumatic brain injury-related deaths--United States, 1997–2007. MMWR Surveill Summ. (2011) 60:1–32. 21544045

[23] Ludvigsson JF Andersson E Ekbom A Feychting M Kim JL Reuterwall C. External review and validation of the Swedish national inpatient register. BMC Public Health. (2011) 11:450. doi: 10.1186/1471-2458-11-450, PMID: 21658213 PMC3142234

[24] Nilsson AC Spetz CL Carsjo K Nightingale R Smedby B. Slutenvårdsregistrets tillförlitlighet. Diagnosuppgifterna bättre än sitt rykte. Lakartidningen. (1994) 91:598, 603–5. 8114596

[25] Wettermark B Hammar N Fored CM Leimanis A Otterblad Olausson P Bergman U. The new Swedish prescribed drug register--opportunities for pharmacoepidemiological research and experience from the first six months. Pharmacoepidemiol Drug Saf. (2007) 16:726–35. doi: 10.1002/pds.1294 16897791

[26] Masnoon N Shakib S Kalisch-Ellett L Caughey GE. What is polypharmacy? A systematic review of definitions. BMC Geriatr. (2017) 17:230. doi: 10.1186/s12877-017-0621-2, PMID: 29017448 PMC5635569

[27] Sjölander A Johansson ALV Lundholm C Altman D Almqvist C Pawitan Y. Analysis of 1:1 matched cohort studies and twin studies, with binary exposures and binary outcomes. Stat Sci. (2012) 27:395–411. doi: 10.1214/12-STS390

[28] Gabbe BJ Simpson PM Cameron PA Ponsford J Lyons RA Collie A. Long-term health status and trajectories of seriously injured patients: a population-based longitudinal study. PLoS Med. (2017) 14:e1002322. doi: 10.1371/journal.pmed.1002322, PMID: 28678814 PMC5497942

[29] Molero Y Sharp DJ D'Onofrio BM Larsson H Fazel S. Psychotropic and pain medication use in individuals with traumatic brain injury-a Swedish total population cohort study of 240 000 persons. J Neurol Neurosurg Psychiatry. (2021) 92:519–27. doi: 10.1136/jnnp-2020-324353, PMID: 33563808 PMC8053342

[30] Ganga A Leary OP Sastry RA Asaad WF Svokos KA Oyelese AA. Antibiotic prophylaxis in penetrating traumatic brain injury: analysis of a single-center series and systematic review of the literature. Acta Neurochir. (2023) 165:303–13. doi: 10.1007/s00701-022-05432-2, PMID: 36529784 PMC9922212

[31] Schindler CR Woschek M Franz J-N Störmann P Henrich D Marzi I. Influence of antibiotic management on microbial selection and infectious complications after trauma. Front Med. (2021) 8:8. doi: 10.3389/fmed.2021.678382 PMC8461005 34568354

[32] Bergold PJ. Treatment of traumatic brain injury with anti-inflammatory drugs. Exp Neurol. (2016) 275:367–80. doi: 10.1016/j.expneurol.2015.05.024, PMID: 26112314 PMC6007860

[33] Dams-O'Connor K Gibbons LE Landau A Larson EB Crane PK. Health problems precede traumatic brain injury in older adults. J Am Geriatr Soc. (2016) 64:844–8. doi: 10.1111/jgs.14014, PMID: 26925541 PMC5021441

[34] Brown-Taylor L Jaramillo C Eapen BC Kretzmer T Gavin LP Cooper T. Accumulation of good intentions: how individual practice guidelines lead to polypharmacy in the treatment of patients with polytrauma. PM R. (2021) 13:1169–75. doi: 10.1002/pmrj.12526, PMID: 33247558

[35] Geller AI Nopkhun W Dows-Martinez MN Strasser DC. Polypharmacy and the role of physical medicine and rehabilitation. PM R. (2012) 4:198–219. doi: 10.1016/j.pmrj.2012.02.012, PMID: 22443958

[36] Levine JM. Common drug interactions following traumatic brain injury. J Head Trauma Rehabil. (2013) 28:151–4. doi: 10.1097/HTR.0b013e31823a5086, PMID: 23466826

[37] Catapano JS Chapman AJ Horner LP Lu M Fraser DR Fildes JJ. Pre-injury polypharmacy predicts mortality in isolated severe traumatic brain injury patients. Am J Surg. (2017) 213:1104–8. doi: 10.1016/j.amjsurg.2016.07.010, PMID: 27596800

[38] Mollayeva T El-Khechen-Richandi G Colantonio A. Sex & gender considerations in concussion research. Concussion. (2018) 3:CNC51. doi: 10.2217/cnc-2017-0015, PMID: 30202593 PMC6094024

[39] Bjork JM Grant SJ. Does traumatic brain injury increase risk for substance abuse? J Neurotrauma. (2009) 26:090330061141047–82. doi: 10.1089/neu.2008-0849 PMC2989860 19203230

[40] Adams RS Larson MJ Corrigan JD Horgan CM Williams TV. Frequent binge drinking after combat-acquired traumatic brain injury among active duty military personnel with a past year combat deployment. J Head Trauma Rehabil. (2012) 27:349–60. doi: 10.1097/HTR.0b013e318268db94, PMID: 22955100 PMC3633079

[41] Olsen CM Corrigan JD. Does traumatic brain injury cause risky substance use or substance use disorder? Biol Psychiatry. (2022) 91:421–37. doi: 10.1016/j.biopsych.2021.07.013, PMID: 34561027 PMC8776913

[42] McDonald S Genova H. The effect of severe traumatic brain injury on social cognition, emotion regulation, and mood. Handb Clin Neurol. (2021) 183:235–60. doi: 10.1016/b978-0-12-822290-4.00011-6 34389120

[43] Schulte MT Hser YI. Substance use and associated health conditions throughout the lifespan. Public Health Rev. (2014) 35:1–27. doi: 10.1007/bf03391702, PMID: 28366975 PMC5373082

[44] Lim XT Ang E Lee ZX Hajibandeh S Hajibandeh S. Prognostic significance of preinjury anticoagulation in patients with traumatic brain injury: a systematic review and meta-analysis. J Trauma Acute Care Surg. (2021) 90:191–201. doi: 10.1097/ta.0000000000002976 33048909

[45] Majdan M Plancikova D Brazinova A Rusnak M Nieboer D Feigin V. Epidemiology of traumatic brain injuries in Europe: a cross-sectional analysis. Lancet Public Health. (2016) 1:e76–83. doi: 10.1016/s2468-2667(16)30017-2 29253420

