# Supplementary material for: Medication utilization in traumatic brain injury patients—insights from a population-based matched cohort study
Source: Front Neurol. 2024 Feb 7;15:1339290. doi: 10.3389/fneur.2024.1339290 (PMC10879380; doi:10.3389/fneur.2024.1339290)
Supplement: Supplementary file 1 [file Data_Sheet_1.pdf]

Supplementary Material

Supplementary Methods, Supplementary Tables 1-6, and Supplementary Figure 1

Supplementary Methods

When comparing medication use in TBI patients to that in the matched population cohort, we applied a fixed-effects model using conditional logistic regression. In this analysis, each matched pair (i.e. the TBI patient and their respective match) was considered a stratum, meaning that the analysis was conducted within each pair. By including fixed-effects for each matched pair (i.e. stratum), we could estimate the odds ratio (OR) of medication use, while controlling for the within-pair variability.

To examine associations between factors related to the patient (i.e. sex) and the injury (i.e. TBI severity), we carried out analyses where we examined TBI patients only. We performed a generalized linear model (GLM) analysis to estimate the risk ratio (RR) for post-TBI medication use where the predictor variable was patient sex and injury severity, respectively. The GLM extends linear regression to handle non-normally distributed variables and quantifies the association between each predictor variable (e.g. sex) and the likelihood of post-TBI medication use. This approach allowed for a comprehensive examination of how patient- and injury-specific factors influenced the probability of post-TBI medication use, while accounting for the non-linear relationship between the predictor variables and the outcome.

Supplementary Table 1. Age at incident TBI stratified by sex.

	**Female TBI patients (n=98,532)**	**Male TBI patients (n=140,893)**
18 to 30	19.5% (19,242)	31.9% (44,874)
31 to 50	20.6% (20,286)	25.1% (35,338)
51 to 70	22.8% (22,419)	23.5% (33,055)
71 and older	37.1% (36,585)	19.6% (27,627)
Median age (IQR)	59 (37, 80)	45 (26, 65)

Supplementary Table 2. Prevalence of medication use 12 months before and 12 months after (stratified on continued use and new use†) the incident TBI date in TBI patients and matched population cohort.

**TBI patients**			
	**12 months before TBI date**	**12 months after TBI date; continued use†**	**12 months after TBI date; new use†**
**Any medication**	64.6% (154,747)	53.2% (127,490)	14.5% (34,664)
**Polypharmacy**	5.2% (12,539)	2.3% (5,463)	3.0% (7,171)
**Gastrointestinal and diabetes medications**	20.2% (48,392)	14.5% (34,681)	7.0% (16,794)
**Cardiovascular medications**	31.6% (75,585)	27.4% (65,573)	5.1% (12,260)
**Genito-urinary medications and sex hormones**	15.1% (36,201)	10.1% (24,242)	4.6% (10,953)
**Systemic hormonal preparations**	10.1% (24,152)	7.1% (16,880)	3.4% (8,135)
**Antibiotics and antivirals**	25.9% (61,996)	11.7% (28,033)	18.3% (43,889)
**NSAIDs and antirheumatics**	14.2% (33,912)	5.7% (13,633)	9.5% (22,782)
**Respiratory system agents**	22.1% (52,791)	12.1% (28,966)	10.1% (24,114)
**Eye medications**	10.0% (23,918)	5.4% (12,905)	5.1% (12,125)
**Matched population cohort**			
	**12 months before TBI date**	**12 months after TBI date; continued use†**	**12 months after TBI date; new use†**
**Any medication**	51.0% (122,070)	41.1% (98,296)	10.4% (123,219)
**Polypharmacy**	2.8% (6,764)	1.2% (3,004)	1.8% (4,253)
**Gastrointestinal and diabetes medications**	13.8% (33,042)	10.1% (24,075)	4.4% (10,577)
**Cardiovascular medications**	24.4% (58,298)	22.1% (52,808)	3.5% (8,351)
**Genito-urinary medications and sex hormones**	12.2% (29,096)	8.6% (20,561)	3.6% (8,727)
**Systemic hormonal preparations**	7.1% (16,915)	5.2% (12,326)	2.4% (5,652)
**Antibiotics and antivirals**	17.3% (41,453)	6.4% (15,418)	10.8% (25,813)
**NSAIDs and antirheumatics**	10.3% (24,633)	4.2% (10,033)	5.9% (14,206)
**Respiratory system agents**	15.6% (37,444)	8.4% (20,085)	7.4% (17,737)
**Eye medications**	7.2% (17,327)	4.1% (9,740)	3.3% (7,855)

† Continued use = Used the medication during the 12 months before the TBI date; New use = Did not use the medication during the 12 months before the TBI; NSAIDs = Non-steroidal anti-inflammatory drugs

Supplementary Table 3. Prevalence of medication use by specific medications; 12 months before and 12 months after the incident TBI date in TBI patients and matched population cohort.

	12 months before		12 months after	
	TBI patients (n=239,425)	Matched population cohort (n=239,425)	TBI patients (n=239,425)	Matched population cohort (n=239,425)
**Any medication**	64.6% (154,747)	51.0% (122,070)	67.7% (162,154)	51.5% (123,219)
**Polypharmacy**	5.2% (12,539)	2.8% (6,764)	5.3% (12,634)	3.0% (7,257)
By specific medications				
**Gastrointestinal and diabetes medications**	**20.2% (48,392)**	**13.8% (33,042)**	**21.5% (51,475)**	**14.5% (34,652)**
Stomatological preparations	4.0% (9,467)	3.0% (7,150)	3.8% (9,200)	3.0% (7,134)
Drugs for acid-related disorders	12.5% (29,929)	7.7% (18,523)	13.8% (33,029)	8.2% (19,706)
Antidiarrheals, intestinal anti-inflammatory/anti-infective agents	2.3% (5,463)	1.3% (3,151)	2.7% (6,359)	1.4% (3,447)
Drugs used in diabetes	5.6% (13,598)	3.9% (9,392)	5.7% (13,598)	4.2% (10,095)
**Cardiovascular medications**	**31.6% (75,585)**	**24.4% (58,298)**	**32.5% (77,873)**	**25.5% (61,159)**
Cardiac therapy	7.3% (17,398)	4.3% (10,403)	7.0% (16,769)	4.4% (10,631)
Antihypertensives	0.4% (831)	0.3% (636)	0.3% (7,690)	0.3% (680)
Diuretics	14.1% (33,754)	9.6% (22,967)	14.2% (33,997)	10.1% (24,107)
Vasoprotectives	1.6% (3,855)	1.1% (2,699)	1.6% (3,883)	1.1% (2,717)
Beta blockers	15.9% (38,788)	12.2% (29,107)	16.2% (38,788)	12.7% (30,301)
Calcium channel blockers	8.1% (19,407)	6.9% (16,512)	8.4% (20,116)	7.5% (17,944)
Agents acting on the renin-angiotensin system	11.2% (26,867)	11.7% (27,981)	15.3% (36,534)	12.7% (30,507)
Lipid modifying agents	11.2% (26,867)	9.3% (22,274)	11.2% (26,920)	9.8% (23,468)
**Genito-urinary medications and sex hormones**	**15.1% (36,201)**	**12.2% (29,096)**	**14.7% (35,195)**	**12.2% (29,288)**
Other gynaecologicals	0.7% (1,606)	0.6% (1,395)	0.7% (1,656)	0.6% (1,462)
Sex hormones and modulators of the genital system	9.9% (23,160)	8.4% (20,136)	9.3% (22,336)	8.3% (1,976)
Genito-urinary medications	5.4% (12,806)	3.6% (8,716)	5.3% (12,780)	3.9% (9,207)
**Systemic hormonal preparations**	**10.1% (24,152)**	**7.1% (16,915)**	**10.5% (25,015)**	**7.5% (17,978)**
Pitituary preparations	0.3% (620)	0.2% (371)	0.3% (612)	0.2% (400)

Corticosteroids for systemic use	5.5% (13,069)	3.5% (8,343)	5.7% (135)	3.7% (8,953)
Thyroid therapy	5.0% (11,935)	3.8% (9,045)	5.2% (12,362)	4.0% (9,599)
Pancreatic hormones	0.1% (137)	0.0% (71)	0.1% (145)	0.0% (74)
Calcium homeostasis	0.1% (110)	0.0% (41)	0.1% (142)	0.0% (52)
**Antibiotics and antivirals**	**25.9% (61,996)**	**17.3% (41,453)**	**30.0% (71,922)**	**17.2% (41,231)**
Antibacterial for systemic use	24.8% (59,312)	16.5% (39,490)	28.9% (69,241)	16.4% (39,191)
Antimycotics for systemic use	1.1% (2,686)	0.7% (1,651)	1.2% (2,881)	0.7% (1,604)
Antimycobacterials	0.1% (150)	0.0% (83)	0.1% (156)	0.0% (72)
Antivirals for systemic use	1.3% (3,036)	0.8% (1,978)	1.3% (3,091)	0.9% (2,070)
**NSAIDs and antirheumatics**	**14.2% (33,912)**	**10.3% (24,633)**	**15.2% (36,415)**	**10.1% (24,239)**
**Respiratory system agents**	**22.1% (52,791)**	**15.6% (37,444)**	**22.2% (53,080)**	**15.8% (37,822)**
Nasal preparations	6.2% (14,927)	5.1% (12,164)	6.0% (14,412)	5.0% (12,028)
Drugs for obstructive airway diseases	7.8% (18,662)	5.3% (12,735)	7.8% (18,573)	5.5% (13,217)
Cough and cold preparations	10.1% (24,138)	7.0% (16,689)	9.7% (23,331)	7.0% (16,733)
Antihistamines for systemic use	6.8% (16,310)	4.1% (9,908)	7.2% (17,317)	4.2% (9,942)
**Eye medications**	**10.0% (23,918)**	**7.2% (17,327)**	**10.5% (25,030)**	**7.4% (17,595)**

NSAIDs = Non-steroidal anti-inflammatory drugs

Supplementary Table 4. Prevalence of medication use in TBI patients during the 12 months before and the 12 months after the TBI date, stratified by sex.

Female TBI patients (n=98,532)		
	**12 months before TBI**	**12 months after TBI**
Any medication	78.7% (77,568)	80.3% (79,090)
Polypharmacy	8.9% (8,323)	8.4% (8,243)
Gastrointestinal and diabetes medications	25.6% (25,204)	26.9% (26,474)
Cardiovascular medications	39.3% (38,740)	40.1% (39,555)
Genito-urinary medications and sex hormones	26.3% (25,863)	25.1% (24,742)
Systemic hormonal preparations	16.2% (15,908)	16.6% (16,360)
Antibiotics and antivirals	33.0% (32,464)	36.1% (35,529)
NSAIDs and antirheumatics	17.1% (16,845)	17.5% (17,249)
Respiratory system agents	27.1% (26,667)	27.1% (26,730)
Eye medications	13.9% (13,642)	14.0% (13,775)
**Male TBI patients (n=140,893)**		
	**12 months before TBI**	**12 months after TBI**
Any medication	54.8% (77,179)	59.0% (83,064)
Polypharmacy	3.0% (4,216)	3.1% (4,391)
Gastrointestinal and diabetes medications	16.5% (23,188)	17.1% (25,000)
Cardiovascular medications	26.2% (36,845)	27.2% (38,278)
Genito-urinary medications and sex hormones	7.3% (10,338)	7.4% (10,453)
Systemic hormonal preparations	5.9% (8,244)	6.1% (8,655)
Antibiotics and antivirals	21.0% (29,532)	25.8% (36,393)
NSAIDs and antirheumatics	12.1% (17,067)	13.6% (19,166)
Respiratory system agents	18.5% (26,124)	18.7% (26,350)
Eye medications	7.3% (10,276)	8.0% (11,255)

NSAIDs = Non-steroidal anti-inflammatory drugs

Supplementary Table 5. Prevalence of medication use in TBI patients during the 12 months after the TBI date, stratified by injury severity.

Inpatient or outpatient treatment of the TBI		
	**Inpatient treatment (n=64,745)**	**Outpatient treatment (n=174,680)**
Any medication	75.0% (48,577)	65.0% (113,577)
Polypharmacy	6.6% (4,243)	4.8% (8,391)
Gastrointestinal and diabetes medications	28.2% (18,248)	19.0% (33,227)
Cardiovascular medications	44.0% (28,459)	28.3% (49,374)
Genito-urinary medications and sex hormones	15.1% (9,777)	14.6% (25,418)
Systemic hormonal preparations	12.6% (8,159)	9.7% (16,856)
Antibiotics and antivirals	32.4% (21,004)	29.2% (50,918)
NSAIDs and antirheumatics	15.7% (10,149)	15.0% (26,266)
Respiratory system agents	23.2% (14,982)	21.8% (38,098)
Eye medications	12.7% (8,240)	9.6% (16,790)
**Polytrauma or TBI without body injuries**		
	**Polytrauma (n=44,509)**	**TBI without body injuries (n=194,916)**
Any medication	72.2% (32,117)	66.7% (130,037)
Polypharmacy	6.2% (2,748)	5.1% (9,886)
Gastrointestinal and diabetes medications	23.5% (10,444)	21.1% (41,031)
Cardiovascular medications	34.6% (15,389)	34.6% (62,444)
Genito-urinary medications and sex hormones	14.6% (6,517)	14.7% (28,678)
Systemic hormonal preparations	11.3% (5,043)	10.3% (19,972)
Antibiotics and antivirals	34.1% (15,192)	29.1% (56,730)
NSAIDs and antirheumatics	18.9% (8,415)	14.4% (28,000)
Respiratory system agents	22.8% (10,147)	22.0% (42,933)
Eye medications	12.0% (5,360)	10.1% (19,670)

NSAIDs = Non-steroidal anti-inflammatory drugs

Supplementary Table 6. Prevalence of medication use in TBI patients during the 12 months before and the 12 months after the TBI date, stratified by age category.

**TBI patients aged 18 to 30 (n=64,116)**		
	**12 months before TBI (n=64,115)**	**12 months after TBI (n=64,115)**
Any medication	44.3% (28,414)	48.1% (30,834)
Polypharmacy	0.6% (377)	0.8% (481)
Gastrointestinal and diabetes medications	5.4% (3,453)	5.8% (3,723)
Cardiovascular medications	1.7% (1,067)	2.0% (1,280)
Genito-urinary medications and sex hormones	13.8% (8,871)	13.7% (8,803)
Systemic hormonal preparations	3.0% (1,923)	3.3% (2,081)
Antibiotics and antivirals	21.8% (13,968)	25.3% (16,248)
NSAIDs and antirheumatics	8.3% (5,312)	10.9% (6,993)
Respiratory system agents	15.5% (9,948)	15.8% (10,140)
Eye medications	3.5% (2,250)	3.9% (2,500)
**TBI patients aged 31 to 50 (n=55,624)**		
	**12 months before TBI (n=55,624)**	**12 months after TBI (n=55,624)**
Any medication	52.0% (28,936)	57.9% (32,176)
Polypharmacy	2.0% (1,135)	2.4% (1,322)
Gastrointestinal and diabetes medications	11.5% (6,381)	12.7% (7,048)
Cardiovascular medications	9.0% (5,029)	10.5% (5,851)
Genito-urinary medications and sex hormones	9.6% (5,328)	9.7% (5,417)
Systemic hormonal preparations	6.0% (3,334)	6.4% (3,558)
Antibiotics and antivirals	22.6% (12,560)	27.6% (15,372)
NSAIDs and antirheumatics	15.3% (8,501)	18.7% (10,423)
Respiratory system agents	21.1% (11,713)	21.7% (12,088)
Eye medications	4.9% (2,698)	5.9% (3,280)
**TBI patients aged 51 to 70 (n=55,474)**		
	**12 months before TBI (n=55,474)**	**12 months after TBI (n=55,474)**
Any medication	71.7% (39,785)	75.9% (42,123)
Polypharmacy	6.6% (3,646)	7.0% (3,877)
Gastrointestinal and diabetes medications	25.2% (13,997)	27.2% (15,074)
Cardiovascular medications	40.6% (22,541)	43.9% (24,331)
Genito-urinary medications and sex hormones	16.1% (8,926)	16.2% (8,959)
Systemic hormonal preparations	11.4% (6,308)	12.1% (6,710)
Antibiotics and antivirals	26.1% (14,469)	30.9% (17,160)
NSAIDs and antirheumatics	19.0% (10,520)	20.2% (11,228)
Respiratory system agents	25.5% (14,136)	26.1% (14,499)
Eye medications	8.4% (4,658)	9.3% (5,129)
**TBI patients aged 71 and over (n=64,212)**		

	12 months before TBI (n=64,212)	12 months after TBI (n=64,212)
Any medication	89.7% (57,612)	88.8% (57,021)
Polypharmacy	11.5% (7,381)	10.8% (6,954)
Gastrointestinal and diabetes medications	38.3% (24,561)	39.9% (25,630)
Cardiovascular medications	73.1% (46,948)	72.2% (46,371)
Genito-urinary medications and sex hormones	20.4% (13,076)	18.7% (12,016)
Systemic hormonal preparations	19.6% (12,587)	19.7% (12,666)
Antibiotics and antivirals	32.7% (20,999)	36.0% (23,142)
NSAIDs and antirheumatics	14.9% (9,579)	12.1% (7,771)
Respiratory system agents	26.5% (16,994)	25.5% (16,353)
Eye medications	22.3% (14,312)	22.0% (14,121)

NSAIDs = Non-steroidal anti-inflammatory drugs

Supplementary Figure 1. Risk ratios of medication use in TBI patients 12 months after the TBI by age category.

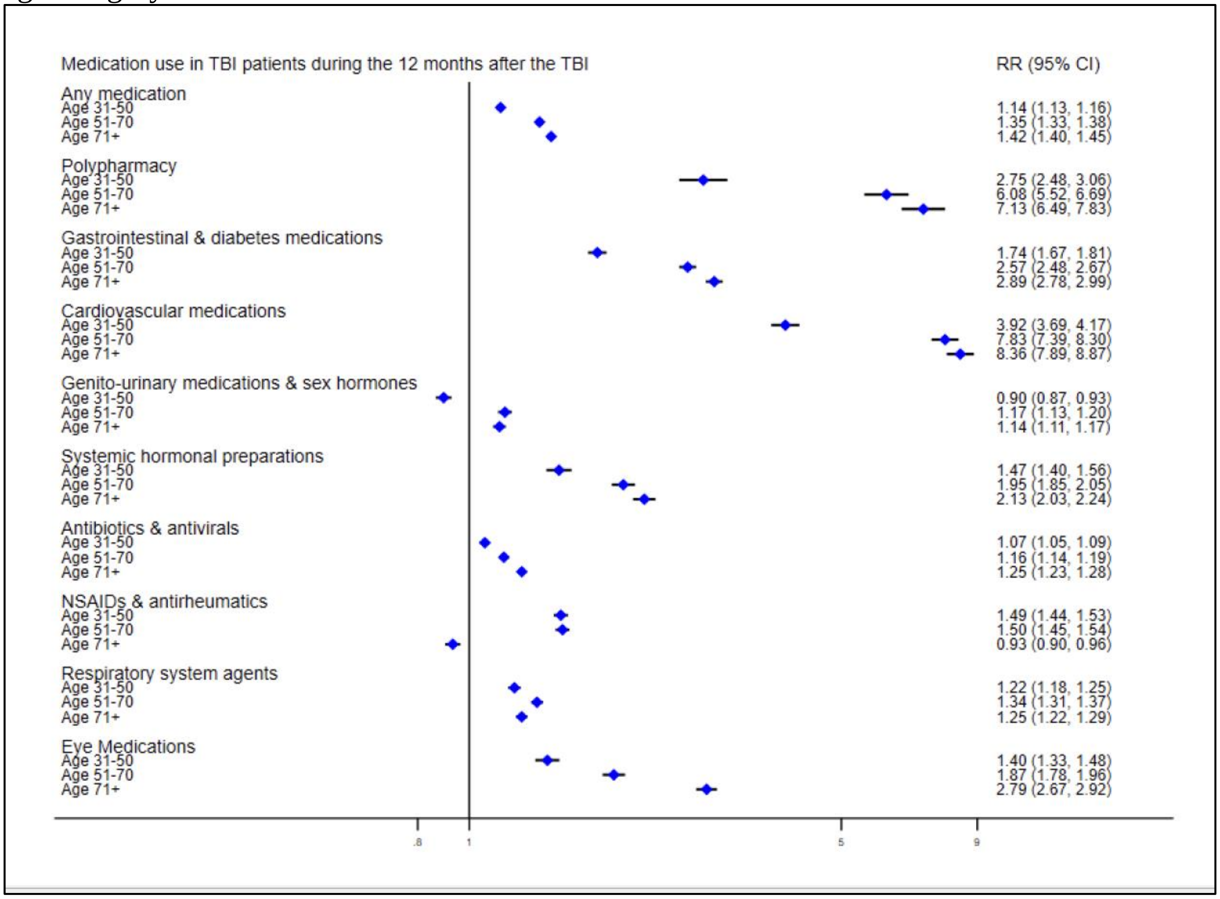

Note: Reference group = TBI patients aged 18 to 30; RR=Risk ratio; NSAIDs = Non-steroidal anti-inflammatory drugs
